# Case Report: Improvement in cognitive functioning following setmelanotide initiation in a patient with Bardet-Biedl syndrome

**DOI:** 10.3389/fendo.2025.1646663

**Published:** 2025-09-19

**Authors:** Menjin Kuk, Jesse Richards, Rachel A. Ross

**Affiliations:** ^1^ Dominick Purpura Department of Neuroscience and Department of Psychiatry, Montefiore Medical Center, Albert Einstein College of Medicine, New York, NY, United States; ^2^ Department of Internal Medicine, The University of Oklahoma Health Sciences, Oklahoma City, OK, United States

**Keywords:** Bardet-Biedl syndrome, melanocortin, setmelanotide, hyperphagia, cognitive function

## Abstract

Bardet-Biedl syndrome (BBS) is a rare genetic condition that results from mutations in a variety of genes crucial for ciliary transport. Consequently, patients with BBS present with a wide array of clinical signs and symptoms that include multiple organ systems. In particular there is a high burden of metabolic disturbances, such as obesity, hyperphagia, and type 2 diabetes, due to the impaired leptin-melanocortin-4 receptor (MC4R) pathway that prevents appropriate activation of MC4R that is normally responsible for signaling hunger and satiety. As such, setmelanotide, an MC4R agonist, has been approved for use to target the obesity and hyperphagia experienced by patients with BBS. Here we report a case of a patient with BBS who was started on setmelanotide for weight management following her BBS diagnosis. One month following treatment initiation, the patient not only endorsed reduced appetite, but also demonstrated a significant improvement in cognitive and affective functioning, as noted in her mental status exam and her performance on the Wechsler Adult Intelligence Scale Fourth Edition (WAIS-IV) tests, when compared to results prior to starting setmelanotide. Although previous studies have reported improved quality of life measures in patients with BBS following setmelanotide initiation, this is the first report of improved cognitive and affective functioning following initiation of the medication, highlighting the need to assess the effects of setmelanotide beyond the metabolic domain in patients with BBS.

## Introduction

Bardet-Biedl syndrome (BBS) is a rare condition that affects multiple organ systems (1). Reports of prevalence appear to be about 1:100,000, with increased incidence in certain geographic regions including Kuwait and Newfoundland (2). Initially reported as examples of cases of Laurence-Moon syndrome, cases of Bardet-Biedl syndrome began to be described in literature starting in 1866 (3). Since then, Laurence-Moon syndrome and BBS have been differentiated into distinct syndromes, albeit with some overlapping characteristics.

Although BBS is a genetic condition with an autosomal recessive inheritance pattern, about 80% of currently identified cases have known genetic etiologies (2). Genetic variants in 28 different genes have been found in association with BBS, especially in the genes that encode the BBSome, an octameric protein complex involved in protein transport in and out of cilia by coating vesicles destined to the cilium. Other genetic variants of BBS also include mutations in proteins that are crucial to the functioning of the BBSome, such as BBS6, BBS10, and BBS12, which form a chaperonin complex to coordinate the early assembly of the BBSome (1, 4).

Given that ciliary function is essential to the function of all cellular systems, mutations in the BBSome or associated proteins have high-reaching consequences in multiple organ systems, leading to the common clinical features seen in patients with BBS. Although each patient with BBS may demonstrate a wide-ranging host of clinical symptoms, the diagnosis of BBS necessitates the presence of a positive genetic test, a certain number of primary criteria, and/or a certain number of secondary criteria, depending on the age of diagnosis. Primary criteria include polydactyly, renal abnormalities, obesity, and retinal dystrophy. Secondary criteria include urogenital abnormalities, neurodevelopmental disability, and anosmia/hyposmia (1, 2).

Notably, there is a high burden of metabolic disturbances in patients with BBS, with 72 - 90% estimated to present with obesity and metabolic dysfunction (1, 3). Studies have found that in BBS1 knockout or deletion models, there is decreased expression of 5HT2C receptors, which contribute to the predominantly food-seeking nature of hyperphagia commonly described in patients with BBS. One survey has demonstrated that about 60% of patients report symptoms that can be categorized as having severe hyperphagia, with the remaining patients categorized as having mild/moderate hyperphagia. In contrast with other conditions that demonstrate hyperphagia and syndromic obesity, such as Prader-Willi syndrome, the hyperphagia observed in patients with BBS has been uniquely characterized to be food-seeking in nature. People with BBS experience extended periods of time of unsatiated satiety, which creates a uniquely obsessive pattern of food-seeking behavior that result in an imbalance between calorie intake and energy expenditure, ultimately driving the early weight gain seen in these patients (5).

Additionally, although the association between the levels of fat-derived hormone leptin and obesity remains unclear in these patients, BBS genes have been shown to affect leptin receptor trafficking, implying a molecular role underlying elevated leptin levels found in patients with BBS. Leptin, in turn, enhances the action of anorexigenic neurons, such as pro-opiomelanocortin (POMC), which decreases appetite by producing an alpha-melanoctye stimulating hormone that activates the melanocortin-4 receptor (MC4R) (1). As such, the impaired leptin-MC4R pathway that hinders appropriate activation of MC4R, as is the case in patients with BBS, can result in increased hunger and reduced satiety that leads to characteristic hyperphagic symptoms.

Given the hypothesized role of an impaired MC4R pathway in patients with BBS, medications that act on that pathway have been of particular interest. Specifically, setmelanotide, an MC4R agonist in the hypothalamus, has been shown to drive a clinically significant decrease in weight or BMI scores, as well as an improvement in reported hunger levels (6, 7). Following a phase 3 trial that evaluated the efficacy of setmelanotide for obesity and hyperphagia in BBS, setmelanotide use has been approved by the FDA to target obesity and hyperphagia for chronic weight management in patients with BBS as of June 2022 (6).

In contrast to such metabolic disturbances that have been better described and examined in patients with BBS, other features of BBS, such as cognitive impairment and psychiatric manifestations, have been less documented and studied. Although intellectual disability appears to be a commonly identified finding in patients with BBS, the exact burden of cognitive impairment varies heavily: there are reports of overall increased prevalence of learning disability but there are varying reports of severity. For example, one study found that the IQ ranges of patients with BBS were in the “borderline range” with the standard score between 70 - 79, and though most patients were evaluated to have social skill deficits, this was attributed to cognitive and sensory differences in patients with BBS, rather than the presence of comorbidities such as severe autism spectrum disorder (ASD) (8). Similarly, another study found that the mean intellectual functioning of patients was 1.5 standard deviations below normal expectations, but the majority did not display a clinically significant intellectual disability (9). With regards to psychiatric comorbidities, studies have found an overall higher burden of psychiatric conditions in patients with BBS, most commonly major depressive disorder, anxiety, and obsessive compulsive disorder, as well as reports of behavioral challenges in childhood described as labile emotional outbursts, with rare reports of externalizing behavior such as aggression (10–12). Relatedly, in the phase 3 trial that evaluated the efficacy of setmelanotide for obesity and hyperphagia, researchers also examined the changes in health-related quality of life in adults and children with BBS following one year of setmelanotide administration. Remarkably, they found clinically meaningful improvements in multiple domains including in psychosocial and physical functioning. Such improvements were supported in a qualitative sub-study, during which patients further endorsed significant improvements in physical and emotional health with increased desires to be more social following setmelanotide treatment (13). As such, although the exact mechanism of action of how setmelanotide may be positively impacting psychosocial functioning in these patients is not yet known, its effects beyond the metabolic domain have been of growing interest. Here we report a case of a patient with Bardet-Biedl syndrome who demonstrated such an outcome, with significant changes in cognitive and affective functioning following initiation of setmelanotide.

## Case description

A 21-year-old female initially presented for clinical neuropsychological evaluation in 2021 to determine the nature and severity of the neuropsychological symptoms she had been endorsing, including frequent affective dysregulation as well as concern for attention deficit hyperactivity disorder (ADHD) and ASD. At this time, patient had not been diagnosed with Bardet-Biedl Syndrome. She has a medical history of hypertension, mixed connective tissue disease, severe obesity (Height 5’11, Weight 217kg, BMI 66.8) with truncal distribution, but was lacking presentation of other primary criteria. She had a psychiatric history of chronic affective dysfunction with reported depression, anxiety, post-traumatic stress disorder, and bipolar disorder. At the time of her initial presentation, her medications included Latuda, Lamictal, fluoxetine, temazepam, hydrochlorothiazide, carvedilol, Plaquenil, Flexeril, and various supplements (cranberry, omega-3, vitamin D). With respect to her social history, the patient graduated from high school with average grades, attempted to attend community college several times but discontinued due to unspecified academic difficulties. She had no history of a learning disability diagnosis or grade repetition. At initial presentation, she was in the process of applying for disability income secondary to affective dysfunction. The patient lived with her spouse of two years and 18-month-old child. Per patient report, she was not independent in her activities of daily living due to anxiety and panic attacks.

## Diagnostic assessment

Neuropsychological testing was conducted, consisting of a mental status exam in addition to assessments of attention/concentration and functioning in the following domains: intellectual, sensory-perceptual, motor, visual constructive, receptive, expressive language, verbal academics, memory, executive, and personality/emotional. In her mental status exam, the patient was documented to have displayed a blunted affect that was reduced in range and intensity, with notably decreased initiative and spontaneity, as well as decreased eye contact.

Overall, the patient demonstrated intellectual and cognitive abilities that were generally within the low average to average range, as shown in **Table 1**. Domains significant for borderline or impaired performances included alternating attention, selective attention and response inhibition, phonemic and semantic verbal fluency, and attentional shift and cognitive speed. The patient’s performance on the Wechsler Adult Intelligence Scale-fourth Edition tests (WAIS-IV) and the Wechsler Memory Scale-fourth edition tests (WMS-IV) were notable for low average performances for the WAIS Processing Speed Index and for 4 of the 5 WMS memory indices (auditory memory, visual working memory, immediate memory, and delayed memory).

**Table 1:** Results of the patient’s neuropsychological test results in 2021, pre-setmelanotide.

Neuropsychological Testing Results, pre-setmelanotide			
Domain	Test	Percentile	Result
Attention and concentration	WAIS-IV FSIQ	53^rd^ percentile	Average
	WAIS-IV Verbal Comprehension Index	50^th^ percentile	Average
	WAIS-IV Perceptual Reasoning Index	63^rd^ percentile	Average
	WAIS-IV Working Memory Index	77^th^ percentile	High average
	WAIS-IV Processing Speed Index	18^th^ percentile	Low average
	WAIS-IV Auditory Working Memory Index	77^th^ percentile	High average
	WMS-IV Visual Working Memory Index	16^th^ percentile	Low average
	Forward digit span of seven	75^th^ percentile	Average
	Backward digit span of six	63^rd^ percentile	Average
	Sequencing of six digits	63^rd^ percentile	Average
	Mental arithmetic	75^th^ percentile	Average
	Sustained attention and discrimination of similar speech sounds	62^nd^ percentile	Average
	Sustained attention and discrimination of similar rhythmic patterns	14^th^ percentile	Low average
	**Alternating attention**	**5^th^ to 16^th^ percentile**	**Borderline to low average**
	**Selective attention and response inhibition**	**6^th^ percentile**	**Borderline**
Sensory-perceptual functioning	Perception of visual details	75^th^ percentile	Average
	Judgment of angular relationships	56^th^ percentile	Average
	Discrimination of similar speech sounds	62^nd^ percentile	Average
	Discrimination of similar rhythmic patterns	14^th^ percentile	Low average
Visual constructive functioning	Reproduction of block designs	63^rd^ percentile	Average
Memory functioning	General fund of verbal information	50^th^ percentile	Average
	WMS-IV Auditory Memory Index	12^th^ percentile	Low average
	WMS-IV Visual Memory Index	37^th^ percentile	Average
	WMS-IV Visual Working Memory Index	16^th^ percentile	Low average
	WMS-IV Immediate Memory Index	19^th^ percentile	Low average
	WMS-IV Delayed Memory Index	18^th^ percentile	Low average
Executive functioning	Associative word reasoning	37^th^ percentile	Average
	Visual measure of analytical reasoning	84^th^ percentile	High average
	Visual puzzles	37^th^ percentile	Average
	Verbal measure of logical and social reasoning	16^th^ percentile	Low average
	Visual measure of logical reasoning	75^th^ percentile	Average
	**Phonemic verbal fluency**	**2^nd^ percentile**	**Impaired**
	**Semantic fluency**	**7^th^ percentile**	**Borderline**
	**Attentional shift and cognitive speed**	**5^th^ percentile**	**Borderline**
	Graphomotor speed and alternating attention	16^th^ percentile	Low average

Highlighted section denotes the testing that was repeated in the 2024 assessment. Bolded texts denotes impaired or borderline percentile results.

For personality and emotional functioning, the patient completed the Personality Assessment Inventory. Results were significant for severely elevated symptoms of generalized anxiety, moderately elevated symptoms of depression, and severely elevated endorsement of somatic and cognitive burdens. The patient also endorsed interpersonal challenges, characterized by aggressiveness/assertiveness, and demonstrated significant characteristics often associated with borderline personality disorder.

With regards to the patient’s concerns for ADHD, she endorsed 73 of the 100 characteristics commonly associated with ADHD, which is markedly elevated. The patient’s mother did not endorse significant symptoms of hyperactivity, impulsivity, or inattention when the patient was a child.

The results of these tests, along with the many symptoms endorsed by both the patient and her mother that were consistent with ASD, the patient’s diagnostic impression at this time was of probable high-functioning ASD, wherein the many emotional, cognitive, and interpersonal difficulties displayed by the patient were attributable to the condition. As such, following the evaluation, the patient was recommended for an initiation of psychotherapeutic treatment targeting symptoms of high-functioning ASD.

## Therapeutic intervention and follow-up

Three years after the neuropsychological testing, in July 2024, the patient was diagnosed with Bardet-Biedl syndrome following a genetic obesity panel that revealed a pathogenic large deletion of the BBS9 gene. Interestingly despite significant neurocognitive abnormalities consistent with known BBS, obesity, reproductive, hepatic, motor and renal issues consistent with Beales Criteria, the patient was not born with polydactyly, which likely contributed to difficulty with initial diagnosis. The patient reported significant history of visual issues but had not been diagnosed with retinitis pigmentosa, so was referred for ophthalmologic evaluation given concern about worsening of visual impairment. The panel also indicated variants of uncertain significance in RPGRIP1L and VPS13B. At this time, the patient was started on setmelanotide to manage her severe obesity (BMI 66), with 0.5mg as her starting dose followed by a slow titration up to a maintenance dose of 3mg over 2 months, which led to weight reduction to BMI of 59.4 (11.1% total body weight reduction).

One month following the initiation of setmelanotide, the patient was re-evaluated by a neuropsychologist at the Marshfield BBS Multispeciality Clinic. At the time of this evaluation, the patient’s setmelanotide dose was 2mg. During this visit, the patient reported significantly improved cognitive functioning attributed to the initiation of setmelanotide, and noted gradual improvement in mental processing speed. The patient also reported that she was now independent in her activities of daily living, including in the management of her finances, medications, and other household affairs. Additionally, although the patient described ongoing difficulties with sleep attributed to probable untreated sleep apnea, she stated improved daily energy levels with the initiation of setmelanotide. She also endorsed reduced appetite, accompanied by a mild laboratory improvement in triglycerides, which is consistent with the known effects of the medication. The patient’s mother and primary caregiver provided collateral history on the patient’s dramatically improved ability to handle activities of daily living such as packing clothes for a trip, going to doctor’s appointments, engaging in conversation, and making eye contact.

At this time, the patient’s mental status exam was significant for expressive speech that was normal in volume, prosody, and articulation, a reported “better” mood,” and a pleasant and cooperative affect. There was no indication of the emotional difficulties revealed on the earlier neuropsychiatric disorder. The WAIS-IV was repeated and indicates some improved outcomes when compared to the same test performed prior to use of setmelanotide (**Table 2**). Notably, there are marked improvements in verbal comprehension and perceptual reasoning scores, with no change in processing speed and FSIQ, and a slightly decreased score on the working memory index. Overall, the patient’s neuropsychological testing at this time demonstrated findings that are largely within normal expectations and general intellectual abilities determined to be within the average range of functioning.

**Table 2:** Results of the patient’s neuropsychological test results one month after initiation of setmelanotide in 2024.

Neuropsychological Test Results, post-setmelanotide			
Domain	Test	Percentile	Result
Intellectual functioning	WAIS-IV FSIQ	63^rd^ percentile	Average
	WAIS-IV Verbal Comprehension Index	58^th^ percentile	Average
	WAIS-IV Perceptual Reasoning Index	84^th^ percentile	High average
	WAIS-IV Working Memory Index	70^th^ percentile	Average
	WAIS-IV Processing Speed Index	18^th^ percentile	Low average

## Discussion

Bardet-Biedl syndrome (BBS) is a rare genetic condition that result from mutations in genes that encode the BBSome or proteins that are essential to the functioning and/or assembly of the BBSome. Because the BBSome has a crucial role in regulating protein transport in and out of cilia, such mutations in, and relating to, this protein complex has cascading consequences that can affect multiple organ systems. As such, patients with BBS display a wide range of clinical signs and symptoms, including cardiac abnormalities, ophthalmologic dysfunction, genitourinary tract anomalies, neurological impairment, and varying degrees of cognitive dysfunction.

Additionally, there is a high burden of metabolic disease in patients with BBS, including obesity and type 2 diabetes. Such symptomology is corroborated by research that has shown the association of BBS gene mutations with impaired leptin receptor trafficking, suggesting a connection between the elevated leptin levels found in patients with BBS and their respective genetic mutations. Given the known downstream effects of leptin in the hypothalamus in ultimately activating the melanocortin-4 receptor (MC4R) that plays a vital role in reducing hunger, the impaired leptin-MC4R pathway has been identified as a site of interest in BBS to aid in the management of appetite regulation (1). Moreover, studies with MC4R knockout mice models have demonstrated differential regulation of food-intake related behavior and cognitive functioning, mediated by expression in the medial prefrontal cortex (mPFC) subregions (14). The mPFC region, in turn, has been commonly cited as the region at which cognition and executive functioning develops, suggesting a connection between the regulation of cognition and MC4R activity in the mPFC region.

This case demonstrates a young patient with BBS who had initiated setmelanotide as primarily indicated by the metabolic burden of her genetic condition. Of note, one month after initiation of the medication, the patient not only endorsed the intended effects of the medication—decreased appetite—but she also demonstrated a positive change in her mental status exam and her ability to independently perform activities of daily living, as compared to her presentation at the time of her neuropsychological testing three years prior. Specifically, she self-reported better management of her mood and affective symptoms, elevated energy levels, and improved ability to independently manage her finances, medications, and household affairs.

**Figure 1:**
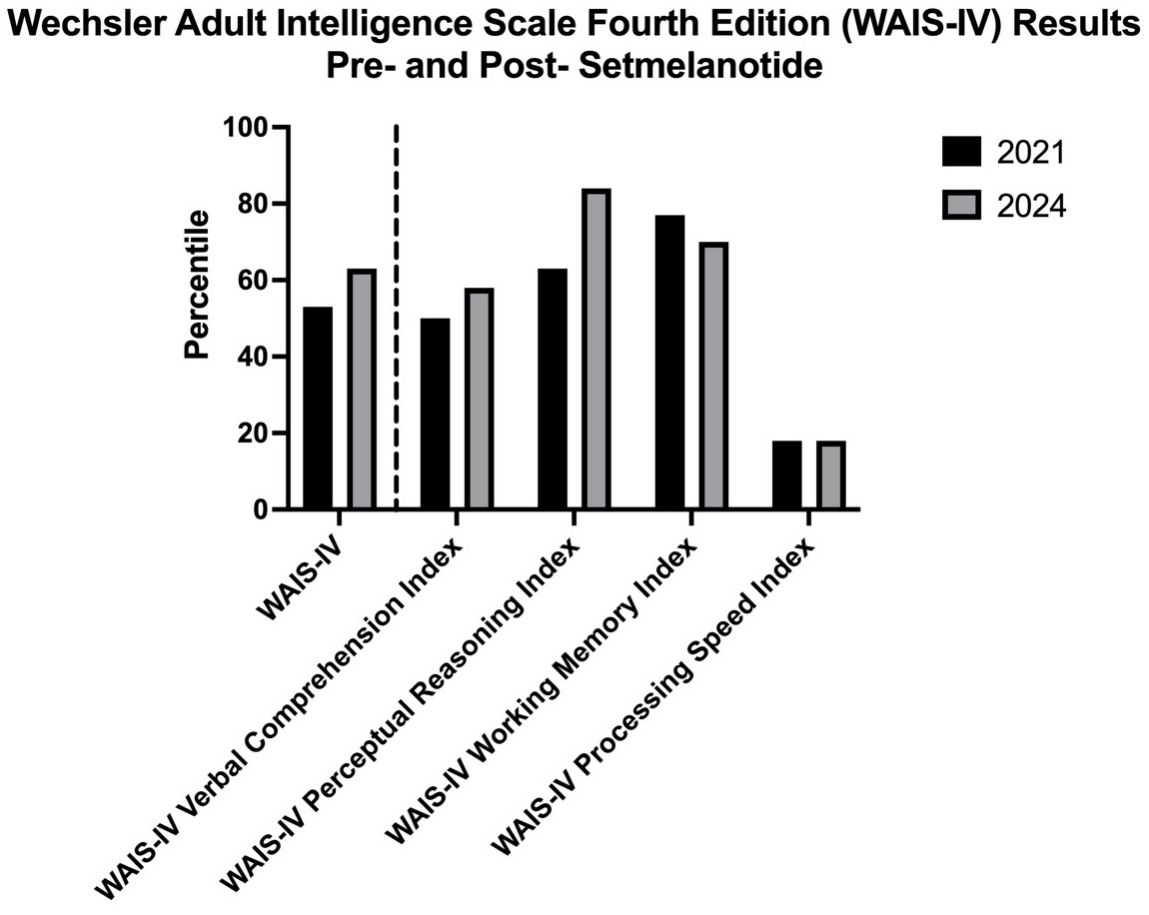
Wechsler Adult Intelligence Scale-Fourth Edition (WAIS-IV) results from testing three years prior to the initiation of setmelanotide compared to the results obtained from testing one month following setmelanotide initiation.

Moreover, the patient demonstrated improved performance in 3 of the 5 main domains in the WAIS-IV when tested one month after initiation of setmelanotide, as compared to her results prior to the medication. As shown in **Figure 1**, the patient’s WAIS-IV FSIQ percentile went up from 53^rd^ to 63^rd^ percentile; WAIS-IV Verbal Comprehension Index went up from 50^th^ to 58^th^ percentile; and WAIS-IV Perceptual Reasoning Index went up from 63^rd^ to 84^th^ percentile. This is surprising as these parameters are thought to be relatively stable across adulthood. Notably, the patient’s performance on the WAIS-IV Working Memory subtest was decreased from the 2021 to the 2024, from the 77^th^ to the 70^th^ percentile. This could be due to issues with attention and anxiety in the context of the test itself—potentially specific to math—or relative fatigue on the day of the assessment, as studies have shown that test anxiety is significantly related to performance on tests that evaluate working memory (15). In particular, it has been demonstrated that math anxiety can negatively influence the working memory subtest results (16). The WAIS-IV Processing Speed was the only subtest that remained the same, at the 18^th^ percentile. Of note regarding both the working memory and processing speed subtests, a study had demonstrated limited efficacy of these two subtests particularly among patients with cognitive impairment (17).

Although studies have shown that repeated exposure to the WAIS tests can superficially increase scores, increases as significant as the changes demonstrated by the patient (increase of 10 percentiles in FSIQ, 8 percentiles in VCI, and 19 percentiles in PRI) have not been documented (18). The marked increase in perceptual reasoning, in particular, is notable given that it is an evaluation of how an individual interprets and thinks with visual information, examining nonverbal reasoning skills that are more fluid. That is, it tests how an individual may be making sense of the world that they perceive visually, an interpretive function, which then informs how they then interact with such a world. Their performance on this subtest can provide insight into the degree to which their interpretations are adapted to the norms, as set forth by the test and by society, thus an executive function commonly attributed to the mPFC region (19, 20). Interestingly, a study had found that individuals with Prader Willi syndrome that demonstrated accurate body image perception had higher PRI scores than individuals who had inaccurate body image perception (21). Though the BMI differences between these two groups were not significant, body image perception critically impacts an individual’s interactions with the world and their interpersonal relationships, thus affecting their psychosocial functioning.

IQ is generally accepted as a stable measure across one’s lifespan, and significant changes in such measures of intellectual functioning after the adolescent years is rare (22). The patient’s demonstrated improvement following the initiation of setmelanotide, in turn, suggests the role of setmelanotide not only with respect to regulating the metabolic effects of BBS, but also the possibility it may impact the emotion, cognition and executive function of patients with BBS. This may underlie the improved quality of life measures seen in the recent larger studies of people with BBS treated with setmelanotide (13), including the improvements in mood and behavior that have recently begun to be reported with setmelanotide initiation (23). More studies using validated measures of executive function testing (i.e. the Brief-2) or other cognitive batteries are needed to better assess the nature of the effect of setmelanotide in the brains of individuals with BBS. It will be important to determine if this effect is lasting and generalizable, as this is the first report in a human of improved cognitive and affective function due to a medication intended to curb appetite.

In conclusion, although setmelanotide has been approved for use in patients with BBS to target obesity and hyperphagia, this patient also demonstrated significant improvements in her cognitive and affective functioning following treatment initiation with the medication. This is the first report of the MC4R agonist having such explicit effects, and it underscores previous studies that have noted improvement in quality of life reported by patients with BBS following setmelanotide treatment. To better understand these effects of setmelanotide, it will be necessary to explore tests that better assess the results shown by this patient and conduct evaluations with a larger group.

## Data Availability

The original contributions presented in the study are included in the article/supplementary material. Further inquiries can be directed to the corresponding author.
